# What Are We Eating? Surveying the Presence of Toxic Molecules in the Food Supply Chain Using Chromatographic Approaches

**DOI:** 10.3390/molecules29030579

**Published:** 2024-01-24

**Authors:** Natalia Casado, Cristina V. Berenguer, José S. Câmara, Jorge A. M. Pereira

**Affiliations:** 1 Departamento de Tecnología Química y Ambiental, Escuela Superior de Ciencias Experimentales y Tecnología, Universidad Rey Juan Carlos, Móstoles, 28933 Madrid, Spain; 2 CQM—Centro de Química da Madeira, Universidade da Madeira, 9020-105 Funchal, Portugal; cristina.berenguer@staff.uma.pt (C.V.B.); jsc@staff.uma.pt (J.S.C.); 3 Departamento de Química, Faculdade de Ciências Exatas e da Engenharia, Universidade da Madeira, 9020-105 Funchal, Portugal

**Keywords:** food safety, food control, contaminants, toxic compounds, food alerts, Rapid Alert System Feed and Food (RASFF), analytical approaches

## Abstract

Consumers in developed and Western European countries are becoming more aware of the impact of food on their health, and they demand clear, transparent, and reliable information from the food industry about the products they consume. They recognise that food safety risks are often due to the unexpected presence of contaminants throughout the food supply chain. Among these, mycotoxins produced by food-infecting fungi, endogenous toxins from certain plants and organisms, pesticides, and other drugs used excessively during farming and food production, which lead to their contamination and accumulation in foodstuffs, are the main causes of concern. In this context, the goals of this review are to provide a comprehensive overview of the presence of toxic molecules reported in foodstuffs since 2020 through the Rapid Alert System for Food and Feed (RASFF) portal and use chromatography to address this challenge. Overall, natural toxins, environmental pollutants, and food-processing contaminants are the most frequently reported toxic molecules, and liquid chromatography and gas chromatography are the most reliable approaches for their control. However, faster, simpler, and more powerful analytical procedures are necessary to cope with the growing pressures on the food chain supply.

## 1. Introduction

Currently, consumers, particularly those in developed and Western European countries, are more aware of the direct impact of food on their health. Consequently, they demand a food industry with a greater degree of information about the products they acquire, which requires this information to be clear, transparent, and reliable [1,2]. Consumers’ perception of food safety risks is recognised in Europe as one of the main pillars directly associated with the subjective perception of food quality. Accordingly, ensuring food safety is an essential aspect of the European food industry to protect the health of consumers, as they have no choice but to trust both manufacturers and farmers when it comes to food production, as well as authorities regarding the enforcement of regulations [2,3]. Hence, in Europe, food safety has become an issue of public concern, implying that the food industry must carry out routine controls throughout the different steps of the food chain to ensure food safety and avoid risks for consumers.

Food safety risks are primarily associated with the unexpected occurrence of contaminants throughout the food supply chain. These contaminants originate from biotic and abiotic sources [4]. Biotic contaminants refer to the occurrence of pathogenic microorganisms, such as bacteria, viruses, fungi, and parasites, whereas abiotic contaminants are those derived from the presence of chemical substances and their derived/transformed products.

Focusing on abiotic contaminants, these chemical substances can be unintentionally present in foods because of the different steps of their production, processing, and transportation. Similarly, they can also be caused by environmental pollution [5]. Many of these chemical substances play an important role in the production and distribution of food, as is the case with pesticides and veterinary drugs, which help improve crop yields and livestock production, as well as reduce production costs and food prices. However, their use can lead to the presence of chemical residues in food, which constitutes a potential risk to the health of consumers. Among the most relevant abiotic contaminants present in food, the following groups can be distinguished:

I. Natural toxins: Toxic chemical substances produced by different organisms. The following categories are included in this group: mycotoxins, plant toxins, and marine toxins.
II. Environmental pollutants: Chemical substances released into the air, water, or soil as a result of industrial or agricultural practices. Pesticides, xenohormones, and veterinary drug residues are also included in this group.
III. Food-processing contaminants: Chemical substances naturally formed in food during industrial processes or cooking.

In the European Union (EU), the authority responsible for evaluating and ensuring food safety is the European Food Safety Authority (EFSA), which evaluates and ensures food safety. This body issues scientific opinions on the different identified food risks. Subsequently, based on the evaluations and reports prepared by the EFSA, the European Commission and EU member states establish European regulations for the correct management of food risks [6]. Thus, within European legislation, there are specific regulations that address the establishment of maximum residue and contaminant limits related to the presence of these chemical substances in foods [7,8,9].

To identify and manage different food risks so that it is possible to protect the health of consumers, the EU has a coordinated food alert system called the Rapid Alert System for Food and Feed (RASFF) [10]. This system allows the rapid exchange of information on food risks between member countries so that immediate measures can be taken, such as removing contaminated products from the market. This tool includes an interactive database called the RASFF window of public access that provides summary information about food alerts notified in the EU (currently limited to 2020 and later) [10]. Thanks to this database tool, consumers, business operators, and authorities around the world can have access to information on recent food recalls and public health warnings in countries linked to this system.

Chromatography is a leading analytical approach for the fast and reliable analysis of molecules in diverse and complex matrices, including foodstuffs. Accordingly, liquid chromatography and gas chromatography cover a wide range of molecules, and their coupling with mass spectrometry makes chromatography a powerful and reliable technique for assessing food composition and safety, enabling the determination of toxic compounds in foods at levels far below those that could threaten human health and well-being. This review aims to provide an overview of food alerts notified in the EU for different categories of abiotic contaminants in recent years (2020–2023) using the information provided by the RASFF window database and highlight the most relevant chemical contaminants responsible for the notification issued. Hence, information on contamination pathways, toxic or adverse effects, and the occurrence of these contaminants in food is included. Examples of the most relevant approaches for determining the presence of these contaminants in food samples using chromatographic techniques are also described. Finally, recent developments and future perspectives for the control and assessment of food safety are discussed.

## 2. Methodology to Review the Food Alerts and Literature

The RASFF window database has been employed to provide a comprehensive overview of food alerts notified in the EU in recent years [10]. The search was limited to the period from January 2020 to June 2023, and it focused only on alerts related to abiotic organic chemical contaminants in food. For this purpose, in the hazard category of the database intended for “risk”, the following filters were selected: “Biocontaminants” OR “Biotoxins” OR “Chemical contamination” OR “Environmental pollutants” OR “Industrial contaminants” OR “Mycotoxins” OR “Natural toxins” OR “Pesticide residues” OR “Residues of veterinary medicinal products”. The data collected were processed and evaluated to determine the main compounds responsible for the alerts in each category. The distribution of the alerts was determined using Microsoft Excel. In addition, to provide a comprehensive overview of the most relevant approaches for determining the presence of these organic chemical compounds in food samples using chromatographic techniques, both Google Scholar and PubMed databases were employed. Accordingly, the following keywords were used: “Food contaminants” OR “Chromatographic analysis”. The results obtained from this search were limited to articles written in English and published in peer-reviewed journals over the last five years. Finally, the abstracts of the retrieved studies were read, and relevant information was used to obtain the data presented in this review.

## 3. Major Classes of Toxic Molecules Reported on Foodstuffs

**Figure 1:**
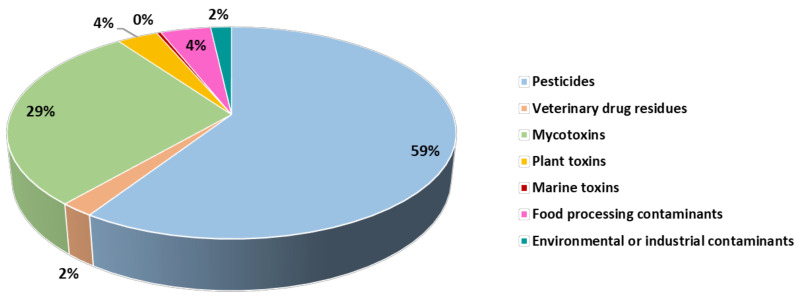
Distribution of food alerts reported between January 2020 and June 2023 in different food contaminant categories (data collected from the Rapid Alert System Feed and Food (RASFF) window, 2023 [10]).

As shown in Figure 1, the highest number of food alerts registered in the EU in recent years was due to the occurrence of pesticide residues in food (almost 60% of the total food alerts issued), followed by the presence of mycotoxins (29% of the total food alerts issued). In contrast, the number of food alerts in the other contaminant categories was significantly lower, with marine toxins having the lowest incidence (0.4% of the food alerts). Each category of chemical contaminants is described in more detail below, highlighting the main compounds responsible for food alerts issued in these categories.

### 3.1. Pesticides

Pesticides are very useful for protecting plants from harmful organisms, including weeds, and improving agricultural production. However, the use of pesticides on crops may lead to the presence of residues of these compounds, including their metabolites, as well as products resulting from their degradation or reactions [11].

**Figure 2:**
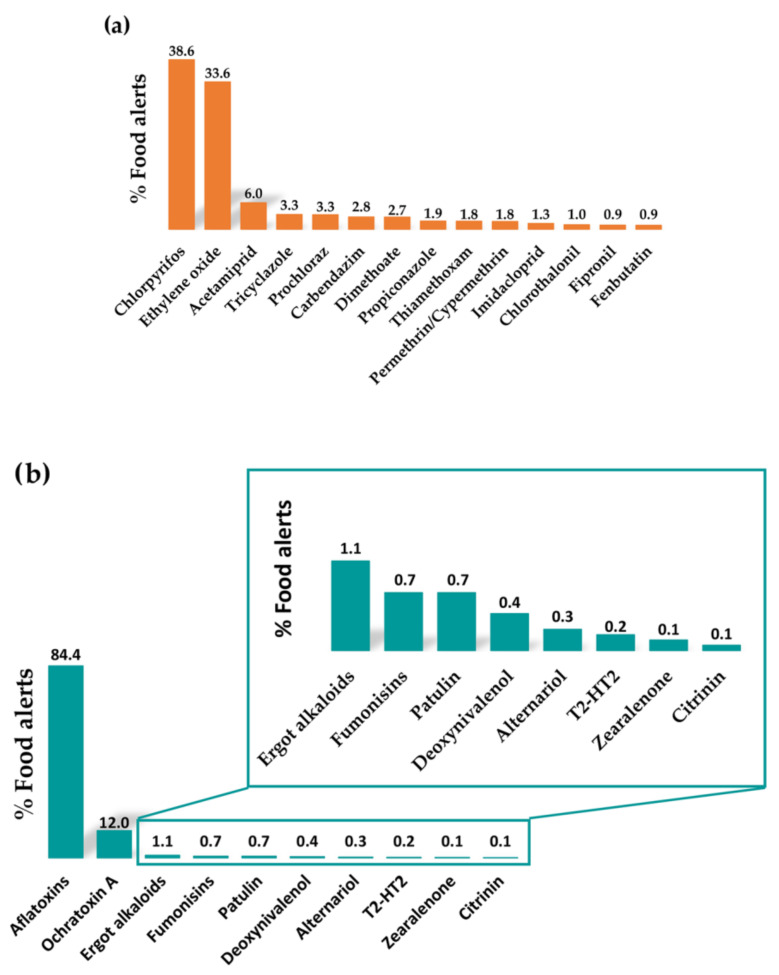

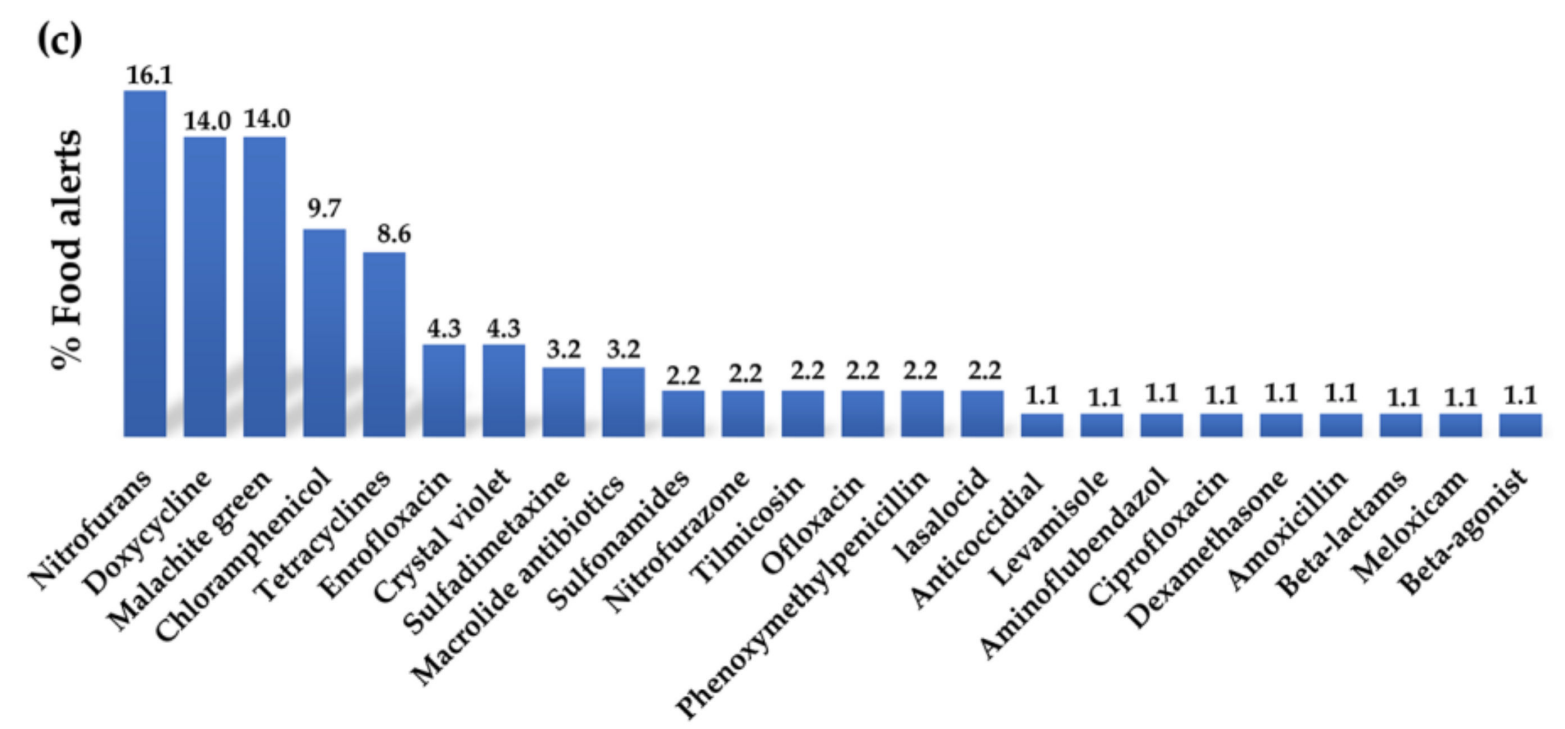
Percentage of food alerts corresponding to the main contaminants detected in the categories of (**a**) pesticides, (**b**) mycotoxins, and (**c**) veterinary drug residues between January 2020 and June 2023 in the Rapid Alert System Feed and Food (RASFF) portal (data collected from RASFF window, 2023 [10]).

Pesticides include herbicides, fungicides, acaricides, plant growth regulators, and repellents. They can be classified according to different aspects such as their use, toxicity, chemical structure, or media lifetime. Based on their chemical structures, it is possible to differentiate organophosphorus pesticides, carbamates, pyrethroids, organochlorines, triazines, benzimidazoles, and nitro compounds [12,13]. Among the recent food alerts reported for the occurrence of pesticides in foods, a huge number have been related to the occurrence of organophosphorus pesticides, such as chlorpyrifos and dimethoate (Figure 2a). It is also worth highlighting the alerts referred to the use of ethylene oxide as a pesticide (Figure 2a), which has been used to sterilise some spices, certain dried herbs, and dried vegetables to control food-borne pathogens such as *Salmonella* and *Escherichia coli*. However, within these food alerts, the occurrence of other pesticides also stands out, such as acetamiprid, prochloraz, tricyclazole, carbendazim, and dimethoate (Figure 2a), which are frequently detected in food products at concerning levels.

Owing to the adverse toxic effects of pesticide residues on human health (Table 1), pesticides cannot be marketed or used without prior authorisation. Accordingly, pesticides are regulated by Regulation (EC) No. 1107/2009 [14], and maximum residue limits (MRLs) for authorised pesticides in foods and feed are included in Regulation (EC) No. 396/2005 [7]. This regulation also contains information on official controls of pesticide residues in foods of plant and animal origin.

### 3.2. Mycotoxins

Mycotoxins are toxic secondary metabolites produced naturally by several species of mould that can grow on food under certain temperature and humidity conditions. Their production is maximal between 24 and 28 °C, whereas their occurrence is lower at refrigeration temperatures [15]. A wide variety of mycotoxins can affect the health of humans and animals, depending on the type of mould that produces them. The most important of these are those produced by mould of the genera *Aspergillus*, *Penicillium*, and *Fusarium*. According to food alerts reported in recent years, the most common are aflatoxins (B1, B2, G1, G2, M1, and M2, produced by *Aspergillus flavus* and *Aspergillus parasiticus*), followed by ochratoxin A (produced by *Aspergillus ochraceus* and *Penicillium verrucosum*), patulin (produced by *Penicillium*, *Aspergillus*, and *Byssochlamys*), fusarium toxins (such as fumonisins, deoxynivalenol, T-2 toxin, HT-2 toxin, and zearalenone, produced by the genus *Fusarium*), and others such as alternariol, citrinin, and ergot alkaloids (Figure 2b).

These mycotoxins can appear at any stage of the food chain in crops contaminated with processed foods. Unprocessed or raw foods, which are more likely to be contaminated with mycotoxins, include cereals, oilseeds, fruits, vegetables, nuts, dried fruits, coffee beans, cocoa beans, and spices. Likewise, these compounds present great technological and thermal stability, so they can resist processes such as grinding and drying as well as subsequent cooking. Consequently, mycotoxins can also be present in processed products, such as cereal-based products (e.g., bread, pasta, and breakfast cereals), beverages (e.g., wine, coffee, cocoa, beer, and juices), baby foods, and even in foods of animal origin (e.g., milk and cheese) [16].

The most common pathway of food contamination with mycotoxins is in the fields during cultivation; however, they can also appear during harvest and storage, or even at several of these stages at the same time. For this reason, owing to their high chemical stability, the most suitable risk management measure to reduce the occurrence of mycotoxins in food, as well as human risk exposure, is by applying the Codes of Hygienic Practice. Currently, there are several Codes of Practice for the prevention and reduction of mycotoxins in some foods, such as patulin and ochratoxin A [17,18,19,20].

The intake of mycotoxins can lead to both acute and long-term adverse effects depending on the type and amount ingested. The acute effects of mycotoxins are primarily gastrointestinal disorders. In contrast, the main disorders derived from long-term exposure to these compounds can be nephrotoxic, hepatotoxic, carcinogenic, genotoxic, and mutagenic, among others. Likewise, some mycotoxins are immunosuppressive, reducing resistance to infectious diseases such as patulin. Table 1 describes the effects of each mycotoxin.

Because of the possible health risks associated with exposure to these compounds by consumers, the EU has a regulation that allows monitoring the occurrence of mycotoxins in food by establishing maximum concentration limits of these contaminants in certain products. Thus, these limits are established in the Commission Regulation (EU) 2023/915 of 25 April 2023 on maximum levels for certain contaminants in food and repealing Regulation (EC) No 1881/2006 [9]. It is also worth noting that the criteria for sampling and analysis of mycotoxins in food have been harmonised in the Commission Regulation (EC) No 401/2006 of 23 February 2006 due to the wide heterogeneous distribution of mycotoxins in foods [21].

### 3.3. Veterinary Drug Residues

Veterinary drug residues can be defined as all pharmacologically active substances, whether active ingredients, excipients, or degradation products and their metabolites, that remain in foods obtained from the animals to which the veterinary drug product has been administered [22]. The health and well-being of food-producing animals are essential to ensure food safety. For this reason, in animal production, a wide variety of drugs are used for therapeutic or zootechnical purposes, because by preventing diseases in animals, public health is also protected.

However, the use of these drugs must be carried out in a responsible and controlled manner to avoid fraudulent practices and abusive use. Otherwise, incorrect administration of these drugs can lead to poor metabolism by the animal, resulting in the occurrence of drug residues remaining in the muscle after slaughter or in the milk after milking [22]. These situations would lead to the entry of these substances into the food chain, posing a serious risk to the health of consumers.

It has been reported that prolonged exposure to these drug residues, even at low doses, can cause different side effects, such as allergic reactions, toxicity, and microbiological, teratogenic, or even carcinogenic effects [23,24,25]. Therefore, controlling veterinary drug residues in food is of utmost importance in the field of food safety. Accordingly, in the EU, substances that are authorised for therapeutic use in animals, as well as those forbidden, are regulated, including MRLs for those allowed (Regulation (EC) No 470/2009 and Commission Regulation (EU) No 37/2010) [8,14]. At the same time, these regulations also establish waiting times that must be respected after administration of the drug before products of animal origin are destined for human consumption.

Despite this, there are a high number of food alerts regarding these compounds (Figure 1). These alerts are notified when quantities of drug residues greater than the MRLs established in legislation are detected or when substances not authorised for veterinary use in animals are used and detected in animal-derived products. The veterinary drug residues most frequently detected in food alerts are antibiotics and antimicrobial substances, such as nitrofurans (e.g., nitrofurazone), sulphonamides (e.g., sulfadimethoxine), and dyes (e.g., malachite green and crystal violet) (Figure 2c). Among the antibiotics, the most common are tetracyclines, particularly doxycycline, followed by chloramphenicol, fluoroquinolones (particularly enrofloxacin), and macrolide antibiotics. The toxic effects of these compounds are summarised in Table 1.

### 3.4. Plant Toxins

**Figure 3:**
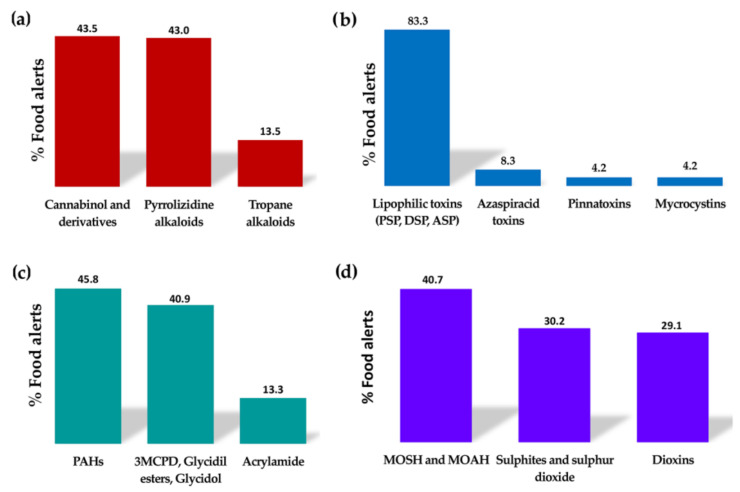
Percentage of food alerts corresponding to the main contaminants detected in the categories of (**a**) plant toxins, (**b**) marine toxins, (**c**) food-processing contaminants, and (**d**) environmental or industrial contaminants within the period from January 2020 to June 2023 in the Rapid Alert System Feed and Food (RASFF) portal (data collected from the RASFF window, 2023 [10]).

Many plants synthesise natural toxins resulting from their secondary metabolism as a defence mechanism; therefore, they are usually highly toxic. Currently, there is increasing awareness about the potential danger that their intake through contaminated food can pose to human health, since until recent years, their occurrence in different foodstuffs has been underestimated [26]. Within this group of contaminants, the food alerts reported in recent years are related to the occurrence of alkaloids, particularly pyrrolizidine and tropane alkaloids (Figure 3a). Generally, these alkaloids are secondary metabolites of plants that grow as weeds in crop fields, leading to contamination of plant-derived products during their production [27]. There are five main families of flowering plants able to produce pyrrolizidine alkaloids: *Asteraceae* (tribes *Senecioneae* and *Eupatorieae*), *Boraginaceae*, *Fabaceae* (genus *Crotalaria*), *Apocynaceae*, and *Orchidaceae* [28]. On the other hand, tropane alkaloids are naturally produced by the following plant families: *Brassicaceae*, *Solanaceae*, *Erythroxylaceae*, and *Convolvulaceae* [29]. The intake of these alkaloids can cause mild disorders (digestive disorders, headache, etc.) to serious situations (neurotoxic, nephrotoxic, hepatotoxic, carcinogenic, mutagenic, and teratogenic effects) or can even be lethal (Table 1). Pyrrolizidine alkaloids can also lead to chronic diseases after long-term exposure, whereas the chronic toxicity of tropane alkaloids has not been demonstrated to date [26]. Both alkaloids have been detected in plant-based products at high concentrations. Pyrrolizidine alkaloids are commonly found in teas, herbal teas, spices, aromatic herbs, honey, and in food supplements. In contrast, tropane alkaloids are mainly found in cereals, pseudocereals, legumes, grains, and derived bakery products. Nonetheless, these alkaloids have also been detected in other products to a lesser extent [26,27,28,29]. To keep consumers’ exposure to these alkaloids as low as possible throughout their diet, the EU has set maximum concentration limits for these toxins in food products. These limits are included in the Commission Regulation (EU) 2023/915 of 25 April 2023 on maximum levels for certain contaminants in food and repealing Regulation (EC) No 1881/2006 [9].

Another toxic compound naturally produced by plants that has caused many food alerts in recent years is the tetrahydrocannabinol (THC) and its derivatives (Figure 3a), which are psychoactive compounds synthesised by the hemp plant (*Cannabis sativa*). In fact, what is present in high concentrations in the crop and in the harvested plant is the precursor of the active molecule, which is then transformed after the application of heat [30]. In the EU, hemp varieties grown and used for food purposes must have a maximum THC content of 0.2% (*w*/*w*) in accordance with Regulation (EU) No. 1307/2013 [31]. Consequently, foods derived from hemp that are authorised to be marketed in the EU are those obtained from the seeds of these hemp varieties, such as oil, hemp protein, and hemp flour. Consequently, it has been estimated that these substances may occur mainly as contaminants in bread, pasta, breakfast cereals, cereal bars, bakery products, teas, beers, and dietary supplements when hemp-derived products are used as ingredients [30]. Nonetheless, small amounts of data are currently available on the actual exposure of the population to these compounds, as well as on the presence of THC in foods of animal origin and on the transfer rate of these compounds from feed to foods of animal origin. The intake of these psychoactive substances mainly affects the central nervous system and is capable of increasing the heart rate even at low doses [30].

### 3.5. Marine Toxins

In general, marine toxins are naturally produced by microalgal species that occur in freshwater and oceans. Consequently, these toxins can contaminate drinking water or accumulate in shellfish and fish when they feed on algae or on other fish that have previously been fed on algae. Nonetheless, some fish species can naturally synthesise toxins (e.g., tetrodotoxins produced by pufferfish) [32].

Regarding the food alerts reported in recent years for marine toxins (Figure 3b), three types of lipophilic toxins stand out: amnesic shellfish poisoning (ASP), mainly related to the presence of domoic acid; diarrhetic shellfish poisoning (DSP), mainly related to the presence of okadaic acid and its derivatives; and paralytic shellfish poisoning (PSP), mainly related to the presence of saxitoxins. The details and effects of the toxins are listed in Table 1.

Intake of these toxins can be a potential hazard to consumers because they can cause several adverse effects (Table 1). However, the main problem with the use of these toxins is that they cannot be reliably eliminated by cooking or freezing because of their high stability [32]. For this reason, the main method to minimise the risk of exposure to these compounds is to limit and control the harvesting area of molluscan shellfish, as well as to remove and discard the viscera in fish.

### 3.6. Food-Processing Contaminants

Industrial or food-processing contaminants are a group of compounds that are naturally generated during food processing and cooking, which have potentially harmful effects on the health of consumers. These contaminants are generated from precursors present in foods (e.g., carbohydrates and amino acids) that undergo chemical changes during processing. These processing procedures include fermentation, drying, smoking, refining, frying, baking, roasting, and grilling [33].

Many foods must be cooked to make them edible and digestible. The application of heat treatment ensures suitable hygiene and microbiological stability of the products. Moreover, thermal processing is crucial for the development of aromas, flavours, and colours in the product. However, food-processing contaminants are also generated through the same chemical reactions that occur during the cooking and preservation procedures of food [33].

Among these contaminants, polycyclic aromatic hydrocarbons (PAHs) are the most relevant. Most of the food alerts collected in this category were caused by the presence of these compounds (Figure 3c). PAHs are chemical compounds composed of carbon and hydrogen atoms that contain two or more aromatic groups. They are mainly formed during incomplete combustion or pyrolysis of organic matter (T > 400 °C). Their occurrence in food can be the result of environmental pollution (industrial activities, heating, forest fires, etc.) or due to food-processing procedures, including smoking, drying, or heating (roasting, barbecues, etc.) [34]. Within PAHs, benzopyrenes have been frequently detected at high levels in food alerts reported in recent years [10]. Cereals, cereal-based products, and fish and fishery products (especially smoked) are the main foods contributing to the total dietary exposure of the population to PAHs. High-fat and high-protein foods prepared on grills or barbecues also contribute to this exposure [34].

Nonetheless, in recent years, many food alerts have emerged for other compounds within the category of processing contaminants, such as acrylamide, 3-monochloropropane diol (3-MCPD), 3-MCPD esters, and glycidyl esters (Figure 3c). These substances have been classified as compounds with possible neoplastic actions, among other effects (Table 1). Acrylamide is formed by the reaction of asparagine (an amino acid) with reducing sugars (mainly glucose and fructose) in the Maillard reaction [34]. Therefore, acrylamide likely appears when cooking or processing starchy foods such as potatoes or cereals at high temperatures (>120 °C) and low moisture levels (i.e., frying, toasting, or roasting). The main dietary sources of acrylamide exposure are coffee, fried potatoes, cookies, crackers, toasts, sliced bread, and certain baby foods.

In contrast, 3-MCPD and its esters, glycidol, and glycidyl esters are generated when high temperatures (>200 °C) are applied to foods high in fat, as in the case of oil refining. These substances were first detected in soy sauce and palm oil. However, it is currently known that the main contributors to total dietary exposure to these contaminants are margarines and derivatives, as well as fats and oils of vegetable origin, followed by bread, pastries, and smoked preserved meat [35].

A common feature of all these food-processing contaminants is their wide distribution in foods, which is an important issue for their monitoring. Thus, consumers’ exposure to these contaminants is transversal and does not generally affect a single specific food or technological process. This means that, to manage the risk of these contaminants, it is necessary to adjust industrial processes and achieve consumer awareness to reduce risk. Currently, the establishment of maximum concentration limits in legislation is the most effective risk management measure to protect consumers from these contaminants. Thus, the Commission Regulation (EU) 2023/915 of 25 April 2023 on maximum levels for certain contaminants in food and repealing Regulation (EC) No 1881/2006 sets limits for PAHs, 3-MCPD and its esters, and glycidil esters [9]. On the other hand, acrylamide has its own regulation (Commission Regulation (EU) 2017/2158), which includes mitigation measures to reduce the presence of acrylamide in foods, reference levels, and a series of codes of good practices that seek to reduce consumer exposure to acrylamide, such as temperature control, cooking time, and the correct selection of raw material measures [36]. Similarly, there is also a Code of Good Practice to prevent and reduce contamination of PAHs in foods produced by smoking and drying procedures [37].

### 3.7. Environmental or Industrial Contaminants

This category includes chemical compounds related to environmental pollution, as they can be released into air, water, or soil, often because of industrial or agricultural activities. Consequently, these substances can enter the food chain at different stages of production, processing, and transport of food products. Many compounds can be included in this group, such as polychlorinated biphenyls (PCBs), dioxins, persistent chlorinated pesticides, brominated flame retardants, and metals (e.g., arsenic, cadmium, lead, and mercury). However, most food alerts reported in recent years in this category have been mainly related to the occurrence of mineral oils (MOH), dioxins, sulphites, and sulphur dioxide (Figure 3d).

MOH is a chemical compound derived from petroleum distillation and refining processes. It can be divided into two main types based on chemical structures: mineral oil saturated hydrocarbons (MOSHs) and mineral oil aromatic hydrocarbons (MOAHs). These compounds can enter the food chain through environmental pollution, the use of lubricants for machinery, release agents, food or feed additives, processing aids, and migration from food contact materials [38]. Accordingly, a large variety of foods may be contaminated with MOH, including oils of vegetable origin, dairy products, cereals and cereal-based products, baby foods, potato chips, legumes, nuts, canned fish, chocolate and chocolate-based products, spices, salads, and ready-to-eat dishes. The potential human health risks derived from MOH intake of MOH varies widely. Currently, MOAHs can act as a genotoxic carcinogen, whereas MOSHs can accumulate in the lymphoid system and damage the liver [38]. However, awareness of the occurrence of these contaminants in food began a few years ago, so it is a relatively recent topic. For this reason, there is currently no regulation regarding assessments and risk management, as well as their monitoring in food products. Nonetheless, data on the presence of these compounds are being collected with the participation of food business operators, manufacturers, processors, and distributors of food contact materials to establish possible regulations in the future.

Dioxins are referred to as polychlorinated dibenzo-p-dioxins (PCDDs) and dibenzofurans (PCDFs). These compounds have no technological or other uses but are generated in many thermal and industrial processes as unwanted and often unavoidable by-products. Thus, they can be generated during the bleaching of paper pulp with chlorine, electrochemical production of chlorine with graphite electrodes, in the textile industry, automobile combustion engines, heating systems, waste incineration, and volcanic eruptions. [39]. Owing to their lipophilic nature, these contaminants tend to accumulate in the fatty tissues of animals. Therefore, the main source of human exposure to these compounds is through diet, mainly through the intake of foods of animal origin with a high fat content. Long-term exposure to contaminants can negatively affect the nervous, endocrine, immune, and reproductive systems. Similarly, these substances may also cause cancer [39]. Nonetheless, these contaminants are usually found in low levels in many foods. In fact, their presence in the environment in Europe has decreased since the 1970s thanks to the efforts of public authorities and industry. In 2001, the EU adopted a strategy to significantly reduce the levels of these compounds in the environment, feed, and foodstuffs to ensure a high level of public health protection [40]. Moreover, the maximum concentration levels for these compounds are currently regulated by Commission Regulation (EU) 2023/915 on 25 April 2023 [9].

Regarding sulphites and sulphur dioxide, these chemical compounds are authorised as food additives in several foodstuffs as preservatives to avoid the growth of bacteria, yeast, and fungi, or as antioxidants to prevent browning processes in fruit and vegetables. Sometimes, they are also used to halt ongoing fermentation during winemaking. Similarly, sulphites are naturally present in our bodies and in some foods, such as onions, rice, apples, cabbages, and wine. However, in some individuals, these substances can cause adverse reactions to the immune system. In 2022, the EFSA carried out a renewed risk assessment for all food additives approved in the EU, concluding that there are gaps in the toxicity data that do not allow confirmation of the extent of certain adverse health effects associated with sulphites and sulphur dioxide [41]. Among these adverse effects, negative effects on the central nervous system were observed, such as a delayed response of nerve cells to stimuli and early signs of nervous system dysfunction. Likewise, it was observed that the estimated intake of these compounds by the population potentially exceeded the safe dose by up to 12.5% in children (3–10 years old) and up to 60% in adults [41]. Therefore, it is important to monitor the levels of these compounds in food products. Accordingly, the food alerts reported in the last few years refer to products that did not comply with the current regulations for these compounds (Regulation (EU) No 1169/2011) [42] or that the presence of these additives was not correctly stated on the food label.

**Table 1:** Origins, contamination pathways, and most relevant toxic effects of the main abiotic organic contaminants.

Compound	Origin and Food Contamination	Toxic Effects	Refs.
Pesticides			
Benzimidazoles	Fungicides used as anthelmintic agents to prevent and treat various types of helminthic parasites (e.g., nematodes and trematodes) in animals used for human consumption. Applied in agricultural products during pre- and post-harvest periods to eradicate pathogens. Resistant to degradation, persisting in the soil for a long time, being easily leached into the water stream, and, thus, entering the food chain and water bodies.	Congenic malformations, teratogenicity, diarrhoea, anaemia, and pulmonary oedemas.	[43,44]
Carbamates	Employed in agriculture as insecticides, nematocides, miticides, molluscicides, and fungicides due to their broad spectrum of biological activity, low cost, and high efficacy. Can enter human bodies through the consumption of contaminated foods.	Reversible inhibitors of acetylcholine esterase enzymes (abnormal function of nerve synapses and neuromuscular junctions). Immune system impairments. Long-term exposure significantly increases the risk of non-Hodgkin’s lymphoma.	[45,46]
Nitro compounds or dinitroaniline pesticides	Broad-spectrum herbicides used for grasses and weed control in the production of fruit trees, nuts, vegetables, green crops, etc. Considered safe agrochemicals because they act specifically on tubulin proteins and inhibit the shoot and root growth of plants.	Acts primarily in plants, but can affect the cellular compartments of animals, causing physiological, metabolic, morphological, and behavioural negative effects in organisms. No toxicity information regarding the potential risk of exposure to non-target organisms.	[47,48]
Organochlorine pesticides	Synthetic organic insecticides used to eradicate pests in agricultural areas, public health sectors, and buildings. Highly resistant to physical, chemical, and biological degradation. Their high lipophilic and slow metabolism profiles facilitate their dissemination through air and aquatic ecosystems, leading to their accumulation in foods, breast milk (human and animal), blood, and adipose tissue of humans, fish, birds, and mammals.	Carcinogenic (liver and prostate cancers), nerve damage, reproductive system abnormalities, diabetes, birth defects, and immune system damage.	[49]
Organophosphorous pesticides	Insecticides applied in fruits and vegetables to kill different types of insects. Can be found in fruits, tea, and water in vestigial amounts.	Potential inhibitors of cholinesterase, nerve system disruption, cerebral palsy, and even death. Cytotoxic, genotoxic, mutagenic, carcinogenic, teratogenic, and immunologic effects.	[50,51]
Pyrethroids	Derived from naturally occurred chrysanthemum esters. Used in agriculture and indoor/outdoor pest control given their relatively low mammalian toxicity, low environmental persistence, and selective insecticide activity. Their widespread use contaminates agricultural products and their residues have been found in many agricultural foodstuffs.	Nausea, vomiting, respiratory depression, mental change, and acute kidney injury.	[52]
Triazines	Herbicides used in agriculture to inhibit or disrupt the normal growth and development of plants and weeds. Their high stability and persistence in the environment led to their bioaccumulation through the food chain, and they have been found in fruit juices, rice, honey, and tea.	High toxicity, endocrine disrupting effects, cancers, and malformations.	[53,54]
Veterinary drug residues			
Beta-lactams	Antibiotics (e.g., penicillin and cephalosporins), mainly used in pig and cattle farms. Often detected in milk, honey, and meat.	Skin infections, diarrhoea, nausea, vomiting, abdominal cramps, endocarditis, pneumonia, osteomyelitis, toxic shock syndrome, and septicaemia.	[55,56]
Dyes (malachite green and crystal violet)	Triphenylmethane dyes used as anti-parasitical agents and fungicides in fisheries and aquacultures.	Mutagenic, genotoxic, and carcinogenic effects.	[57]
Fluoroquinolones	Synthetic antibiotics used to treat cattle, chickens, and fish. Broad antibacterial activity. Residues are often found in foodstuffs due to incomplete metabolisms.	Drug resistance of pathogenic bacteria.	[58]
Macrolide antibiotics	Antibiotics used in medical care, fish breeding, and poultry raising. Bacteriostatic effect through the inhibition of bacterial protein synthesis. Residues can be found in several surface and drinking water sources.	Endocrine disruption and hormonal action.	[59]
Nitrofuranzone	Synthetic antimicrobial substances to treat bacterial and protozoan infections in livestock, aquaculture, bees, and feed additives.	Carcinogenic, teratogenic, mutagenic, and inhibition of DNA synthesis in bacterial and mammalian cells.	[60]
Sulphonamides	Synthetic antibiotics used in veterinary bacteriostasis. Unabsorbed sulphonamides can enter the environment through urine and faeces, contaminating soil and water. Cause adverse effects on aquatic organisms. Can accumulate in the human body through the food chain.	Drug resistance, allergies, teratogenesis, cancer, and damage to the urinary system.	[61]
Tetracyclines	Used to treat bacterial infections and promote animal growth (livestock husbandry). Residues found in animal-derived foods (e.g., milk, honey, and pork). Difficult to degrade and remove.	Liver injury, tooth yellowing, bacterial resistance, allergic reaction, and intestinal flora disorder.	[62]
Plant toxins			
Cannabinoids and derivatives	Nonpsychoactive phytocannabinoid and hemp derivatives have been increasingly used in food (beverages, chocolates, coffee, and tea) to address pain, anxiety, depression, insomnia, tension headaches or migraines, post-traumatic stress disorder, nausea, cancer, allergies or asthma, multiple sclerosis, chronic obstructive pulmonary disease, Parkinson’s disease, and Alzheimer’s disease.	May be linked to the neurodevelopment of the adolescent brain. Could impact neural and brain development.	[63,64]
Pyrrolizidine alkaloids	Produced as a defence mechanism against herbivores and insects. Identified in plants from the families of *Asteraceae*, *Fabaceae*, *Boraginaceae*, *Orchidaceae*, and *Apocynaceae*. The main contamination source seems to be the accidental co-harvesting of weeds containing pyrrolizidine alkaloids. Other contamination paths include horizontal natural transfer through the soil and animal feed with producing plants of these contaminants.	Hepatotoxic, pneumotoxic, genotoxic, and carcinogenic effects.	[28,65,66]
Tropane alkaloids	Produced by different plants, mainly from the *Solanaceae* family. Can be found anywhere in the plant, including seeds, fruits, flowers, leaves, and stems, which can lead to cross-contamination due to fast and mechanical harvesting.	Anticholinergic compounds, avoiding the binding of acetylcholine with the muscarine receptor. Causing tachycardia, muscle spasms, mydriasis, delirium, and eventually death.	[29,67]
Marine toxins			
Amnesic shellfish poisoning (ASP)	Produced by some species of unicellular algae from the genus *Pseudonitzschia*. Main toxic compound is domoic acid. Associated with the consumption of molluscan shellfish because of their occurrence in the viscera of Dungeness crab, tanner crab, red rock crab, and anchovies.	In the early stages, usually intestinal distress is experienced. Severe ASP can cause a facial grimace or chewing motion, short-term memory loss, and difficulty breathing. Death can occur.	[34]
Diarrhetic shellfish poisoning (DSP)	Produced by some species of unicellular algae from the genera *Dinophysis* and *Prorocentrum*. Main toxic compounds are okadaic acid and its derivatives. Associated with the consumption of molluscan shellfish.	Diarrhoea, nausea, vomiting, moderate-to-severe abdominal pain and cramps, and chills. No known fatalities have occurred, and total recovery is expected within three days, with or without medical assistance.	[34]
Paralytic shellfish poisoning (PSP)	Produced by some species of unicellular algae from the genera *Alexandrium*, *Pyrodinium*, and *Gymnodinium*. Main toxic compounds are saxitioxins. Associated with the consumption of molluscan shellfish because of their occurrence in viscera of mackerel, lobster, Dungeness crabs, tanner crabs, and red rock crabs.	In the early stages, there is numbness and a burning or tingling sensation of the lips and tongue that spreads to the face and fingertips, causing a general lack of muscle coordination in the arms, legs, and neck. Severe cases resulted in respiratory paralysis and death.	[34]
Mycotoxins			
Aflatoxins	Produced by the fungi *Aspergillus*, which grows in foodstuffs (e.g., peanuts, mouldy corn, soybeans, rice, and other grain and oil crops) stored in warm and humid conditions. Predominantly found in Asia and Africa.	Aflatoxin B_1_ is the most toxic aflatoxin. Genotoxic, immunotoxic, teratogenic, carcinogenic, and mutagenic toxic effects.	[68,69]
Alternariol	Metabolite of *Alternaria* fungi found in cereal-based raw materials, vegetables, and fruit. *Alternaria* species are highly adaptable to environmental conditions and can grow and produce toxic secondary metabolites at low temperatures.	Allergic rhinitis, chronic rhinosinusitis, and asthma. Genotoxic effects cause inhibition of DNA relaxation, stimulate DNA cleavage activities, and induce double-stranded DNA breaks.	[70,71,72]
Citrinin	Antibacterial mycotoxin produced during the fermentation of rice with moulds of the genus Monascus (“red mould rice”) and food storage and cultivation. Used for meat preservation and food colouring.	Nephrotoxic, hepatotoxic, and genotoxic effects.	[73,74,75,76]
Deoxynivalenol	Trichothecene mycotoxin (*Fusarium graminearum* and *Fusarium culmorum*) detected in cereal grains such as wheat, corn, barley, rice, oats, sorghum, rye, and fresh agro-products.	Nephrotoxic, hepatotoxic, and genotoxic effects.	[77]
Ergot alkaloids	Mycotoxins produced mainly by fungi of the *Claviceps* genus, which parasitise the seeds of living plants, such as rye, triticale, wheat, oat, and barley. They replace the grain with fungal structures known as sclerotia that contain alkaloid substances and can be harvested together with cereals or grass. Can be found in cereal-based foods and feed.	Intoxications and illnesses (e.g., ergotism, characterised by abdominal pain, vomiting, burning sensation of the skin, insomnia, and hallucinations).	[78]
Fumonisins	Generated by *Fusarium proliferatum* and *Fusarium verticillioides.* Can be found in feedstuff, foodstuff, and crops. Produced in dry and warm conditions. Can also result from insect stress.	Oesophageal cancer, estrogenicity, immunotoxicity, teratogenicity, mutagenicity, carcinogenicity, and inhibition of ceramide synthase leading to hindered sphingolipid biosynthesis and disrupted biological membranes.	[79]
Ochratoxin A	Mainly produced by fungi of the genera *Aspergillus* and *Penicillium.* Commonly found in food and raw agricultural products (e.g., cereals, corn, peas, coffee, cocoa, beer, wine, grapes, dairy, and meat products of animals consuming contaminated grains).	Hepatotoxic, carcinogenic, nephrotoxic, genotoxic, and embryotoxic effects, as well as teratogenicity, immunotoxicity, mutagenicity, and testicular toxicity.	[77,80]
Patulin	Produced during foodstuffs storage by *Penicillium* species (e.g., *P. expansum*). Found in various fruits (e.g., apples and apple products—juice, pies, conserves) and baby food. Unstable in grains, cured meats, and cheese. Can be degraded during food processing or storage, generating other toxic compounds.	Immunosuppressive, immunotoxicity, and genotoxicity effects and inhibits DNA synthesis. Exposure has immunological, gastrointestinal, and neurological effects.	[81,82]
T-2 and HT-2 toxins	Type-A trichothecenes formed by *Fusarium* fungi. Lipophilic toxins stable to heat and acidic conditions and, thus, not destroyed during normal food processing or digestion. Easily absorbed through skin, gut, and pulmonary mucosa. Can be found in maize, oats, wheat, barley, rye, rice, walnut, and tomatoes.	Gastric and intestinal lesions, hematopoietic and immunosuppressive effects, anorexia, lethargy, nausea, suppression of reproductive function, corneal injury, hypotension, shock, and potent inhibitors of protein synthesis. Immunosuppressive and dermatotoxic effects can lead to necrosis and haemorrhage of the intestinal mucosa.	[83]
Zearalenone	*Fusarium* secondary metabolite, also known as F-2 toxin. Common contaminant of foodstuffs (e.g., corn products, cereal crops, wheat, corn, and other grains).	Digestive system dysfunction, neurotoxicity, reproductive toxicity, embryotoxicity, carcinogenicity, immunotoxicity, genotoxicity, induced oxidative stress, and apoptosis.	[84]
Food-processing contaminants			
Acrylamide	Produced by asparagine amino acid and reducing sugar under Millard’s reaction (nonenzymatic browning). Found in heat-treated foods during roasting, baking, and frying processes above 120 °C (e.g., potato chips, French fries, rice, coffee and coffee products, tea, and baked foods).	Neurotoxic and genotoxic effects. Damage the nervous system.	[85,86]
Glycidol and glycidyl esters	Occur during the vegetable oil refining process. Subsequently, the refined vegetable oil is used during food thermal processing and in frying and baking processes. Glycidyl ester can be found in cereal products (bakery, cereal, and biscuits), roasted coffee, infant formulas, potatoes and snack foods, spreads, and fried food (domestic frying processes).	Genotoxic carcinogen due to the release of glycidols from their parent esters after ingestion into the gastrointestinal tract.	[87,88]
Polycyclic aromatic hydrocarbons (PAHs)	Aromatic compounds with two or more benzene rings composed of carbon and hydrogen. Produced by food thermal processing during the incomplete combustion or high-temperature cracking of organic substances. Commonly found in air, water, soil, and sediment, in the form of complex mixed solids at ambient temperature. Residues have been detected in edible oils, dairy products, fruits and vegetables, coffee, baked products, chocolate, cereals, aquatic seafood, and meat products (smoked and roasted meats).	Interfere with the normal function of biological cell membranes and membrane-related enzyme systems. Symptoms include vomiting, nausea, diarrhoea, skin inflammation and redness, kidney and liver damage, decreased immunity or immunosuppression, blood cell rupture, and congenital disabilities. Genotoxic, mutagenic, and carcinogenic effects.	[89,90]
3-MCPD and its esters	Process contaminants detected in refined vegetable oils, fish oils, oil-based foodstuff, cereal-based food, infant formula, and soy sauces, among other foodstuffs.	Neurotoxic, genotoxic, and carcinogenic effects.	[87,91]
Environmental or industrial contaminants			
Dioxins and furans	Persistent organic pollutants can be released through natural processes and atmospheric emissions of industrial processes (e.g., waste incineration and thermal and combustion-related activities) and traffic. Enter the food chain when animals eat contaminated plants, mainly meat and dairy products, fish, and shellfish.	Neurodevelopmental impairment, damage to the immune system, and interference with hormones acting as endocrine disruptors. Carcinogenic, immunotoxic, and adverse effects on reproduction and development.	[92,93]
Mineral oil hydrocarbons (MOHs)	Petrogenic-originated mixtures composed of many isomers mainly derived from crude oil. Also, produced synthetically from coal, natural gas, and biomass. Contamination occur through transfer from the food packaging surface, environmental pollution, or intentional use during food production.	Genotoxic carcinogens that can damage DNA and may cause cancer.	[94]
Sulphites and sulphur dioxide	Preservatives used in food and beverage production to prevent browning or oxidation during preparation, storage, and distribution. Used to inhibit nonenzymatic and enzymatic browning in pharmaceutical and food industries. Acts as an antioxidising and antibacterial agent in brewing industries.	Vitamin deficiency, hypersensitivity, allergic diseases, anaphylactic reactions, dermatitis, urticaria, flushing, hypotension, abdominal pain, and diarrhoea.	[95,96,97]

## 4. Overview of the Main Chromatographic Approaches Used to Assess Food Safety

Regardless of the target contaminants and food matrices analysed, LC is consistently the preferred chromatographic approach to be used and reported in the last five years (2018–2023, as reviewed in [98]). This is often preceded by sample preparation using commercial SPE, QuEChERS, among other procedures [99]. Sample preparation and extraction is an important step that will dictate the success of the chromatographic analysis, and for this reason, specific information about the extraction procedures followed in each of the selected reports is also presented in Table 2. There have been some improvements to these standard protocols for the extraction of the mycotoxin citrinin in cereals, food supplements, and red yeast rice using molecularly imprinted polymers as sorbents in the SPE procedure [75] or the extraction of the toxin okadaic acid in clams using magnetic SPE [100]. Regarding QuEChERS, successful downscale ability to extract tropane alkaloids in leafy vegetables is noteworthy [67]. Deep eutectic solvents (DES) have also been employed in the liquid–liquid microextraction of organophosphorus and pyrethroid pesticides from fruit juices and teas [50,52]. Another interesting report on the analysis of PAHs in nutritional supplements containing omega-3 and fish oil involved fabric sorbent-phase extraction (FPSE) [89]. The application of covalent organic frameworks (COFs) and metal organic frameworks (MOFs) to obtain sorbents with augmented retention capabilities has been successfully explored in recent years, particularly for the extraction of antibiotics from different foodstuffs (reviewed in [101,102]). Overall, from the reports compiled in Table 2, it is clear that, despite the improvements and innovations introduced in the extraction procedure, MS detection is essential to obtain a better analytical performance. However, as observed in the determination of citrinin in red yeast rice, the use of improved extraction protocols (MISPE) partially compensates for the lack of MS detection systems, clearly improving the analytical performance of the methodologies reported using the HPLC-FLD architecture [73,75]. GC-MS is used less frequently than LC for the analysis of food contaminants, because its range of applications is limited to volatile and semi-volatiles compounds. A derivatisation procedure to obtain volatile molecules is sometimes possible; however, this can make the procedure longer and more prone to errors, resulting in poorer analytical performance. Nevertheless, it is worthwhile to refer to the use of GC-MS/MS to detect dioxins and furans in different meats, salmon, and fish oils [92], or more recently, to detect genotoxic carcinogens of vegetable origin in infant formulas and elderly milk powders [87].

**Table 2:** Toxic molecules reported in foodstuffs and the methodology used to assess their safety found in the literature in the last five years.

Compound	Sample	Extraction Method	Analysis	Results (LODs/Recoveries)	Ref.
Pesticides					
Imidacloprid, acetamiprid, clothianidin, and atrazine	Fruits and vegetables	QuEChERS: 5 g sample, 5 mL ACN; 0.6 g MgSO_4_, and 0.2 g PSA	LC-MS/MS	0.08–141 μg/kg/70–110%	[103]
Organophosphorus pesticides	Juices, water, tomato, cucumber, and honey samples	75 mL sample; nanocomposite comprising metal-organic framework MIL-101(Cr), and graphene nanopowder	GC-MS	0.005–15.0 µg/kg/84–110%	[51]
Organophosphorus pesticides	Vegetables	30 min sonication of 4 g homogenised samples mixed with 8 mL ACN; collect the filtrate; repeat three times; combine and evaporate (50 °C N_2_ stream); redissolve (1 mL acetone); MSPE: add 25 mg Fe_3_O_4_@COF@Zr^4+^ to the sample solution; 30 min vortex; discard supernatant; elute (1 mL acetone; 8 min US); 0.22 μm filtration	GC-FPD	0.7–3.0 μg/kg/87–121%	[104]
Organophosphorus pesticides (phosalone and chlorpyrifos)	Red grape juice and sour cherry juice	10 mL sample; DES-UALLME: choline chloride/4-chlorophenol (408 μL)	HPLC-UV	0.070–0.096 ng/mL/87.3–116.7%	[105]
14 organophosphorous pesticides	Fruits and vegetables	2 g sample; SPME: N-doped C-(C_3_N_4_@MOF) fibre coating	GC–MS	0.23–7.5 ng/g/82.6–118%	[50]
Pyrethroids (transfluthrin, fenpropathrin, fenralerate, ethofenprox, and bifenthrin)	Tea beverages and fruit juices	5 mL sample; DES-DLLME: Hexafluoro-isopropanol-based hydrophobic DES (0.15 g)	HPLC-DAD	0.06–0.17 ng/mL	[52]
Neonicotinoids	Water	2 mg MOFs + 1 mL NEOs standards; 5 min incubation; centrifugation (14,000 rpm, 2 min); 500 μL MeOH ultrasonic elution; vacuum evaporator, 100 μL mobile phase solubilisation	LC-MS	0.02–0.1 ng/mL	[106]
Veterinary drug residues					
52 veterinary drug residues	Mutton or leg meat	5 g sample; QuEChERS: modified with reduced graphene oxide-melamine sponge (r-GO@MeS)	UPLC–MS/MS	LOD: 0.02–2.0 μg/kg LOQ: 0.05–5.0 μg/kg/63.7–109.5%	[107]
103 veterinary drug residues	Milk and dairy products	5 g liquid milk or 1 g milk powder; QuEChERS with dispersive solid phase: 100 mg C_18_ and 300 mg anhydrous sodium sulphate	UPLC-MS/MS	LOQ: 0.1–5 μg/kg (milk) and 0.5–25 μg/kg (milk powder)/>60%	[108]
Beta-lactams, quinolones, sulphonamides, and tetracyclines	Fish, poultry, and red meat	1 g sample; SPE: 5 mL ACN	LC-MS/MS	LOD: 0.3–15 µg/kg, LOQ: 0.8–45.3 µg/kg/82–119%	[109]
Sulphonamides	Pork, milk, and water	100 mL sample loaded through the TPB-DMTP-COF column; washing (3 mL water); drying; elution (8 mL MA); drying (N_2_ flow); eluent re-dissolved (1.0 mL ultrapure water)	LC–MS/MS	0.5–1.0 ng/L	[110]
Malachite green and crystal violet	Hairtail fish	5 g sample; dSPE: NiO/ZnO-coated carbon microspheres, 3 mL 3:7 MeOH–H_2_O, 4 mL 9:1 MeOH	UPLC-UV	0.50 μg/L (malachite green) and 0.35 μg/L (crystal violet)	[57]
8 nitrofurans	Muscle, milk, eggs, honey, and casings	2 g sample; hydrolysis and derivatisation, followed by ethyl acetate extraction	UHPLC-MS/MS	93.5–127.5% recovery	[60]
Doxycycline	Chicken claws	2 g sample; extraction with 5 mL 5% TCA	UHPLC−MS/MS	5 μg/kg/80–110%	[111]
Estrogens	Milk and cosmetics	5 mL milk + perchloric acid (100 μL, 10% *v*/*v*); homogenisation and centrifugation (3 min 10,000 rpm); supernatant pH adjusted to 4 (NaOH, 1 M); 0.45 μm filtration; lotion centrifugation (10 min 10,000 rpm); supernatant pH adjusted to 4 (HCl 1 M); 0.45 μm filtration; add 40 mg MILs + 0.275 g NaCl; 5 min shaken 1500 rpm; recover MILs; 500 µL ACN elution	HPLC-UV	5–15 ng/mL/98.5–109.3%	[112]
Biotoxins					
Ergot alkaloids and their epimers	Oat-based foods and food supplements (bran, flakes, flour, grass, hydroalcoholic extracts, juices, and tablets)	QuEChERS: 1 g sample; 4 mL ACN and 5 mM ammonium carbonate (85:15, *v*/*v*); dSPE: 150 mg C_18_:Z-Sep+ (1:1); residue reconstituted with 750 µL MeOH 50% (*v*/*v*), 0.22 µm nylon membrane filter	UHPLC–MS/MS	LOQ: 3.2 μg/kg/89.7–109%	[78]
Lipophilic marine toxins (yessotoxins, dinophysistoxins, okadaic acid, azazspiracids, and spirolides)	Fresh and processed shellfish	100 g sample; QuEChERS: 2 mL MeOH/ethanol/isopropanol; dSPE: 50 mg graphene oxide/ 100 mg MgSO_4_	UPLC-MS/MS	LOD: 0.10–1.47 μg/kg LOQ: 0.32–4.92 μg/kg/85–117.4%	[113]
Staphylococcal enterotoxin type A (SEA)	Cow’s milk	25 g sample, clean up: pH control (pH 3.5 ± 0.5 + 5 M HCl; pH 7.5 ± 0.1 + 5 M NaOH) and TCA precipitation (20% TCA solution); protein denaturisation (5 mL 100 mM Tris-HCl, pH 8.5, 7 M guanidium hydrochloride + 10 mM EDTA); enzymatic digestion and desalting: trypsin digestion (1:100 (*w*/*w*)), 1% formic acid acidification, desalting with a GL–Tip styrene-divinylbenzene	LC–MS/MS	LOQ: 10 µg/kg/70–120%	[114]
Okadaic acid	Clams	MSPE: 2 g samples + 9 mL MeOH, mix; clean-up: 3 mg Fe_3_O_4_@TaTp dispersed in 200 μL MeOH, extraction (5 mL blank seawater containing okadaic acid) and derivatives incubated with Fe_3_O_4_@TaTp; rinse with 200 μL ultrapure H_2_O, 90% MeOH desorption (50 μL); extraction: 5 mg Fe_3_O_4_@TaTp dispersed in 200 μL MeOH, extraction with 1 mL reconstituted solution of shellfish samples spiked with okadaic acid and derivatives incubated with Fe_3_O_4_@TaTp; rinse with 200 μL ultrapure H_2_O, 200 μL ACN desorption; 0.22 μm nylon filtration	LC-MS/MS	0.5 pg/mL (seawater) and 0.04 µg/kg (shellfish)	[100]
Pinnatoxin-G	Mussels	2 g mussel tissue; 9 mL methanol; 2.5 mL methanolic extract hydrolysed with 313 µL 2.5 M NaOH; neutralised with 313 µL 2.5 M HCl; 0.22 µm filtration	LC–MS/MS	LOD: 0.1 µg/kg LOQ: 0.4 µg/kg/62–110%	[115]
Biocontaminants					
Tropane alkaloids	Leafy vegetables	0.1 g sample; µQuEChERS: 150 mg MgSO_4_, and 25 mg PSA	HPLC-MS/MS	LOQ: 2.2–2.3 ng/g/82–110%	[67]
Histamine	Cheese and cured meat products	10 g sample; 100 mL HNO_3_ (0.1 mol/L); ultrasonication (15 min, 35 kHz, 40 °C)	IC-PCD	0.15 mg/kg/91.3–116.9%	[116]
	Mackerel canned fish	5 g sample; 20 mL perchloric acid 0.2 M; SPE: 0.5 g cationic exchange resin; column derivatisation: ortho-phthalaldehyde (0.1 mL), and 2-mercaptoethanol	HPLC-UV	LOD: 1.8 mg/kg LOQ: 5 mg/kg/98–99%	[117]
7 cannabinoids	Hemp products: seeds, cannabis-infused beer, energy drink, chocolates, roasted coffee and tea	Beer and energy drink (30 mL): SPE (1 mL hydrochloric acid 0.1 M/ 2 mL MeOH); chocolates, hemp seeds, and hemp tea (0.02 g): UAE (10 mL MeOH)	LC-MS	LOD: 2.19 ng/mL LOQ: 6.59 ng/mL/70.0–110%	[63]
21 pyrrolizidine alkaloids	Oregano samples	0.2 g sample; QuEChERS: 150 mg MgSO_4_ and 25 mg PSA	UHPLC-MS/MS	LOD: 0.1–7.5 µg/kg, LOQ: 0.5–25 µg/kg/77–96%	[65]
14 pyrrolizidine alkaloids and pyrrolizidine alkaloid N-oxides	Teas and weeds	1 g sample; 0.1 M sulphuric acid; SPE: 1% formic acid, and 5 mL MeOH/4 mL MeOH + 0.5% ammonium hydroxide	UHPLC-MS/MS	LOD: 0.001–0.4 μg/kg LOQ: 1–5 μg/kg/ 68.6–110.2%	[66]
Mycotoxins					
Citrinin	Red yeast rice	LLE: 30 mg sample, 2 mL H_2_O–acetone 2:3 (V/V)	HPLC-FLD	4 mg/kg/109.9%	[73]
	Nutraceutical green tea	SPE: 1 g sample, sorbent zirconia-coated silica and PSA	UHPLC-HRMS	LOQ: 0.2 μg/kg/97%	[74]
	Cereals, food supplements and red yeast rice	MISPE: 0.5 g sample, molecularly imprinted polymer	HPLC-FLD	550–1105 μg/kg/75.6–90.7%	[75]
Alternariol, alternariol monamethyl ether, tenuazonic acid, tentoxin, deoxynivalenol, and patulin	Cherry tomato, lettuce, and pakchoi	SPE: 1 g sample, HLB SPE cartridges (hydrophilic N-vinyl pyrrolidone and lipophilic diethyl benzene)	UHPLC-MS/MS	LOD: 0.05–3.0 μg/kg LOQ: 0.2–10.0 μg/kg 81.1–116%	[70]
19 mycotoxins	Lotus seeds	QuEChERS: 1 g sample, 5 mL ACN 80% (*v*/*v*), 150 mg C18, and 150 mg MgSO_4_ anhydrous	UHPLC-MS/MS	0.1–15.0 μg/kg/84.6–96.4%	[76]
17 mycotoxins	Edible nuts	QuEChERS: 5 g sample, 10 mL ACN-formic acid (99.9/0.1 (*v*/*v*)); dSPE-EMR-lipid: 0.4 g NaCl, and 1.6 g anhydrous MgSO_4_	LC-MS	0.05–5 μg/kg/ 75–98%	[118]
Chemical and industrial contaminants					
PAHs	Nutritional supplements containing omega-3 and fish oil	FPSE: sol–gel phenyl/polydimethylsiloxane (PDMS)-coated FPSE membranes back-extracted with ACN	HPLC-UV	LOD: 2.16–2.50 ng/mL LOQ: 6.50–7.50 ng/mL/63.2–102.3%	[89]
Sulphites	Herbal teas	dSPE: ACN and 0.1% acetic acid + 10 mM ammonium acetate	UPLC-MS/MS	0.51–12.1 μg/kg/83.8–102.7%	[95]
Sulphur dioxide	Stir-fried foods, dried fruits, preserved fruits, ginger, and shredded squid	1 g sample; 25 mL NaOH 0.4 mM; derivatisation: 2 mL sample disodium hydrogen phosphate and potassium dihydrogen phosphate buffer (pH 5.5)/2.50 mL phthalaldehyde and 1.5 mL ammonium acetate	HPLC-FLD	LOD: 0.2 mg/kg LOQ: 0.7 mg/kg/82.32–105.08%	[97]
Acrylamide	French fries, bakery biscuits, and branded biscuits	1 g defatted sample; 10 mL H_2_O; 0.5 mL Carrez I and Carrez II solutions; filtration (0.45 μm cellulose acetate syringe filter paper)	HPLC-DAD	LOD: 3.733 ng/μL LOQ: 11.045 ng/μL/98–110%	[85]
	Coffee and coffee products	QuEChERS: 0.5 g roasted coffee or 2.5 g ready-to-drink (brewed) + 5 mL dichloromethane; SPE Carb/SCX/PSA cartridge; acrylamide residue transformed to 2,3-dibromoacrylamide (acrylamide-Br_2_) by KBr derivatisation (1 mL 15% (*m*/*v*)) and potassium bromate (100 μL 1.7% (*m*/*v*)) at acidic conditions (70 μL 10% (*v*/*v*) sulphuric acid); 0.22 μm PTFE filtration	UPLC-MS/MS	Roasted and instant coffees: LOD: 1.2 μg/kg LOQ: 4 μg/kg; Ready-to-drink coffees: LOD: 0.24 μg/kg LOQ: 0.8 μg/kg/ 99.3–102.2%	[86]
Polychlorinated dibenzo-*p*-dioxins and furans	Boiled eggs, crab meat, beef, sheep liver, herring, cod liver, salmon, and fish oil	Dichloromethane/n-hexane (1:1, *v*/*v*); acidic silica gel (44% sulphuric acid) to remove lipids and polar interfering substances	GC-MS/MS	LOQ: 0.005–0.101 ng/mL (GC-APCI-MS/MS) and 0.006–0.201 ng/mL (GC-EI-MS/MS)	[92]
Glycidyl esters	Infant formulas and elderly milk powders	Transesterification by automation: 0.5 g sample, 2 g anhydrous sodium sulphate, and 2 mL distilled H_2_O; 10 mL hexane: ethanol (2:1, *v*/*v*); residue re-dissolved with 400 μL isooctane	GC-MS/MS	LOD: 0.8 μg/kg/91.7–111.3%	[87]
Sodium iron chlorophyllin and sodium copper chlorophyllin	Candies	0.1 N hydrochloric acid (5 mL), ultrasonication (50 °C, 10 min), dilution to 20 mL (MeOH); vortex mixing, centrifugation (10,000 rpm, 10 min), filter upper layer (0.2 μm)/HPLC-MS	UHPLC-MS	LOD/LOQ: 1.2; 4.1 mg/kg (SIC); 1.4; 4.8 mg/kg (SCC),	[119]

Legend: ACN—acetonitrile; C-(C_3_N_4_@MOF)—metal organic framework-based porous carbon; COF—covalent organic framework; DAD—diode-array detection; DES—deep eutectic solvent; DLLME—dispersive liquid–liquid microextraction; dSPE: dispersive solid-phase extraction; EMR—enhanced matrix removal; GC-APCI-MS/MS – gas chromatography-atmospheric pressure chemical ionization-tandem mass spectrometry; GC-EI-MS/MS – gas chromatography coupled with electron impact–tandem mass spectrometry; FPSE—fabric phase sorptive extraction; GC-FPD—gas chromatography equipped with flame photometric detector; GC-MS—gas chromatography–mass spectrometry; GC-MS/MS – gas chromatography tandem mass spectrometry; HCl—hydrochloric acid; HNO_3_—nitric acid; HPLC—high-performance liquid chromatography; HPLC-DAD—high-performance liquid chromatography with diode-array detection; HPLC-FLD – high performance liquid chromatography with fluorescence detector; HPLC-UV—high-performance liquid chromatography with ultraviolet detection; IC-PCD—ion chromatography post column derivatisation; LC—liquid chromatography; LC-MS—liquid chromatography–mass spectrometry; LLE—liquid–liquid extraction; LOD—limit of detection; LOQ—limit of quantitation; MeOH—methanol; MgSO_4_—magnesium sulphate; MILs—magnetic ionic liquids; MISPE: molecular imprinting solid-phase extraction MOF—metal organic framework; MSPE: magnetic solid-phase extraction; MS/MS—tandem mass spectrometry; NaCl—sodium chloride; NaOH—sodium hydroxide; PSA: primary secondary amine; PAHs—polycyclic aromatic hydrocarbons; QuEChERS—quick, easy, cheap, effective, rugged, safe; SPE: solid-phase extraction; TCA—trichloroacetic acid; TPB-DMTP-COF—triphenylbenzene-dimethoxyterephthaldehyde-COFs; UAE—ultrasound-assisted extraction; UALLME—ultrasound-assisted liquid–liquid microextraction; UHPLC—ultra-high-performance liquid chromatography; UHPLC-HRMS — ultra-high-performance liquid chromatography-high resolution mass spectrometry; μ—miniaturised.

### 4.1. Recent Developments and Future Perspectives in the Control of Food Safety Using Chromatographic Approaches

In recent decades, there has been growing recognition of the adverse effects of human activity on the environment, which has prompted an increase in the search for more environmentally friendly analytical methodologies, including chromatography. Large-scale multiresidue methods that enable the simultaneous analysis of a large number of compounds can reduce the number of necessary analyses. Rizzo et al. [120], for instance, proposed an analytical platform using salting-out-assisted liquid–liquid extraction of aqueous extracts combined with ultra-high-performance liquid chromatography–high-resolution tandem mass spectrometry for the screening of 88 pyrrolizidine alkaloids in food matrices with a high risk of contamination. In turn, Steiner, et al. [121] developed an LC-MS/MS-based multiclass approach for the accurate quantification of >1200 biotoxins, pesticides, and veterinary drugs in complex feeds. This approach was challenged with more than 130 real compound feed samples, providing the first insight into the co-exposure of animal feed to agricultural contaminants. A reliable and efficient method for analysing 302 targeted contaminants in catfish muscle was also developed and validated. This method was designed to detect pesticides and their metabolites at US regulatory levels as well as other lipophilic pesticides and environmental contaminants, including PAHs, PCBs, PBDEs, and other flame retardants. The sample preparation was based on the QuEChERS extraction technique. The extracted sample was divided and analysed using UHPLC-MS/MS for 128 analytes after filtration and low-pressure (LP) GC-MS/MS for 219 analytes after an automated robotic micro-SPE clean-up [122]. Another remarkable example was reported by Fialkov et al. [123], who designed an LP GC-MS system capable of achieving good separation with full analysis cycle times of less than one minute. This was accomplished by combining low-pressure GC-MS with low thermal mass resistive-heating for rapid temperature ramping and cooling of the capillary column. This method was successfully applied to replicate the EPA Method 8270 using a complex mixture of 76 semivolatile compounds, which are typically quantified using conventional GC-MS. This approach has great potential for the rapid analysis of PAHs in food samples [123]. Another methodology using GC-MS/MS has been devised to analyse 209 pesticides and persistent organic pollutants (POPs) in non-target wildlife animal liver tissues. This technique requires only 100 mg of liver tissue and allows for the detection of multiple residues in each sample [124].

Micellar liquid chromatography is a green chromatographic approach that is notable for its minimal requirement for organic modifiers, such as acetonitrile and methanol, and ease of recycling the mobile phase. This results in a reduction in excess solvents. Micellar liquid chromatography has a wide range of applications, including, but not limited to, the analysis of antibacterial substances, melamine, biogenic amines, plant protection products, flavonoids, and peptides in various biological matrices, such as milk, eggs, tissues, honey, and feed [125]. The assessment of the more environmentally friendly profile of micellar liquid chromatography was further investigated by Mohamed and Fouad [126], who proposed three alternative HPLC methods for determining the levels of sulfadiazine and trimethoprim in bovine meat and chicken muscles. After thorough evaluation using the GAPI, NEMI, and analytical eco-scale, it was concluded that micellar liquid chromatography demonstrated superior environmental performance.

#### 4.1.1. Multidimensional Chromatography

Methods involving two consecutive chromatographic separations, hereby considered multidimensional (MD) chromatography, have great potential by combining the resolution power of the chromatographic approaches taken individually. However, these formats require sophisticated and expensive instrument configurations and expertise that can be challenging to achieve [127]. Nevertheless, advancements in LC, such as increased orthogonality, separation power, sensitivity, and the ability to hyphenate with more powerful MS detectors, are boosting foodomic investigations, leading to an increase in the number of applications, including food contaminant analyses [128,129]. In this respect, MD-LC has gained significant popularity over the past few years for separating non-volatile analytes from complex matrices. Conventional one-dimensional LC cannot resolve potential co-elutions or minimise matrix effects, which can hinder accurate quantitative analysis. However, coupling MD-LC with MS results in a notable enhancement of the separation power or peak capacity, owing to increased selectivity and sensitivity, making it a valuable tool for many applications, such as the quantification of mycotoxins [127]. Mycotoxins are major contaminants in agricultural products, and several other MD-LC approaches have been developed for their analysis, such as a multi LC-LC coupled with the ESI–MS/MS method for the determination of seven mycotoxins in beer [130], or a 2D-LC HRMS method for the simultaneous monitoring of 70 regulated and emerging mycotoxins in Pu-erh tea [131]. Other notable examples of this approach include enhanced analytical capacities for the analysis of aromatic biogenic amines using 2D heart-cutting sequential injection chromatography [132] and the determination of dangerous compounds in milk and colostrum by coupling MD-LC with HR-MS [128].

Online LC-GC streamlines the sample preparation process, thereby saving time and improving the sensitivity and reliability of analysis. This MD system integrates sample preparation in the first dimension (LC) and analysis in the second dimension (GC). The LC dimension has a high sample capacity, whereas the GC dimension offers a high separation efficiency and the ability to utilise various detectors, including MS. A recent automatised interface, named TOTAD, has been proposed to eliminate manipulation errors and offer different operation modes that enhance analytical performance (e.g., the ability to inject or transfer large volume fractions regardless of the eluent used) [127]. Another promising development in this field is a compact 2D GC system that incorporates microfabricated columns and a nanoelectromechanical system resonator as the detector. This system is eco-friendly, portable, and capable of ultra-fast chromatographic separation, making it suitable for a range of applications where size, weight, power, and speed are critical, including real-time and on-site food safety assays [133].

#### 4.1.2. Miniaturisation of Chromatographic Architectures

The scaling down of traditional macroscale systems, including conventional chromatographic architectures, offers several advantages, including a substantial reduction in the consumption of reagents, samples, and energy, as well as faster and more cost-effective analytical processes, resulting in shorter analysis times. Furthermore, such systems are more prone to efficient automation, resulting in higher throughput and multiplexing [134]. The miniaturisation of GC methodologies involves less energy consumption, whereas the same strategy applied to LC results in a reduction in solvent consumption [135]. One example of these approaches is the bubble-in-drop (BID) microextraction of carbamate pesticides followed by GC-MS analysis. This method utilises only 1.00 μL of the extraction solvent and an air bubble volume of 0.40 μL to determine carbamates in water with good recovery rates, low limits of detection, and high enrichment factors [136].

Another path involves the development of new architectures, such as that proposed by Liao et al. [137], employing a cellular design that simultaneously performs the partial separation of analytes during the sampling process. The authors assayed this promising progressive cellular architecture in microscale GC using a range of polar and nonpolar analytes with wide molecular weights and vapour pressure variations, including alkanes, alcohols, aromatics, and phosphonate esters. Under these conditions, separations within 12 min at a column temperature of 63–68 °C and resolutions greater than two for any two homologues that differ by one methyl group were achieved [137].

Regarding LC developments in this field, nano-LC offers several advantages that align with green chemistry principles, such as reduced flow rate and solvent consumption, resulting in a lower environmental impact and cost of analysis. Common HPLC stationary phases, including C18 sorbents with particle sizes of 3–5 µm or smaller, can be used in nano-LC methods. Additionally, nano-LC methods have been found to have several advantages when applied to pesticide analysis compared to other types of LC, including requiring fewer sample preparation steps and achieving greater sensitivity. Given the increasing regulatory requirements for detecting contaminants, there is a strong demand for more capable analytical methods, and nano-LC has the potential to provide better analytical performance than other chromatographic methods [138]. Moreno-González et al. [139] reported a remarkable example of the nano-LC potential for determining pesticide residues in specific parts of bee specimens. The method developed allows for the extraction of useful information from specific bee parts of individual specimens and provides pseudo spatially resolved chemical information about pesticide contamination [139]. The presence of pyrrolizidine alkaloids in honey, tea, herbal tinctures, and milk was also determined with increased sensitivity and reduced solvent consumption using nano-LC-MS with high-resolution Orbitrap mass spectrometry [140].

#### 4.1.3. Portable Chromatography Solutions and Chromatography-on-Chip

Polycyclic aromatic hydrocarbons (PAHs) are classified as priority hazardous substances because of their carcinogenic properties and potential threats to public health. There are strict regulations in place to prevent their release into the environment, but these regulations are not consistently enforced due to the lack of a reliable field-testing procedure. To address this challenge, Chatzimichail et al. [141] developed a hand-portable system capable of separating, identifying, and quantifying PAHs. The developed system incorporates an HPLC and a spectrally wide absorption detector, which can identify all 24 PAHs on the priority pollutant list of the United States Environmental Protection Agency [141]. In addition, an alternative chipHLPC device using fluorescence and electrospray mass spectrometry (ESI-MS) was used to obtain a rapid and on-site separation of four PAHs [142]. Another microfluidic chromatography detection system was used to measure the concentrations of saccharin sodium (SAC) and acesulfame potassium (Ace-K) in 16 commercial food samples, providing rapid detection of artificial sweeteners in food [143].

Unconventional print and media technologies have also been applied in the field of chromatography, resulting in the creation of a compact, all-in-one LabToGo system. This emerging field, referred to as office chromatography (OC), employs additive manufacturing for the 3D printing of functional components, as well as open-source hardware and software. For example, the analysis of steviol glycosides in Stevia leaves yielded results comparable to those obtained through traditional methods, while the bioanalytical screening of water samples enabled the evaluation of potential health and environmental risks [144].

## 5. Conclusions

This paper offers a comprehensive overview of the presence of toxic molecules in the food chain and the crucial role of chromatography to ensure food safety. It highlights the importance of stringent regulations and continuous monitoring to safeguard consumer health. The application of various chromatographic techniques, such as liquid chromatography, gas chromatography, and mass spectrometry, in the assessment of food safety has demonstrated their essential roles in the detection and quantification of contaminants and toxins in food and environmental samples. Emergent improvements in the field of chromatography are essential to obtaining faster and more robust chromatographic analysis. Overall, the review underscores the critical importance of chromatographic methods in addressing the challenges of toxic compounds in the food chain and their indispensable role in ensuring food safety and consumer well-being.
